# Muscle Injuries: A Brief Guide to Classification and Management

**Published:** 2014-09-01

**Authors:** Nicola Maffulli, Angelo Del Buono, Francesco Oliva, Alessio Giai Via, Antonio Frizziero, Michele Barazzuol, Paola Brancaccio, Marco Freschi, Stefano Galletti, Gianfranco Lisitano, Gianluca Melegati, Gianni Nanni, Ghito Pasta, Carlo Ramponi, Diego Rizzo, Vittorino Testa, Alessandro Valent

**Keywords:** muscle injury, sports medicine, classification, rehabilitation

## Abstract

Muscle injuries are frequent in athletes. Despite their high incidence, advances in clinical diagnostic criteria and imaging, their optimal management and rehabilitation strategies are still debated in literature. Furthermore, reinjury rate is high after a muscle lesion, and an improper treatment or an early return to sports can increase the rate of reinjury and complications. Most muscle injuries are managed conservatively with excellent results, and surgery is normally advocated only for larger tears. This article reviews the current literature to provide physicians and rehabilitation specialists with the necessary basic tools to diagnose, classify and to treat muscle injuries. Based on anatomy, biomechanics, and imaging features of muscle injury, the use of a recently reported new classification system is also advocated.

## Introduction

Muscle injuries are frequent in high demand sports, accounting for 10 to 55% of all acute sports injuries [1]. The muscles and muscle groups more frequently involved are the hamstrings, rectus femoris, and the medial head of the gastrocnemius. Although the diagnosis is usually clinical, imaging tools are often advocated to better identify the extent and site of lesion, the relevant prognostic factors predictive of recovery time, return to pre-injury sport activity, and risk of recurrence [2]. Many different treatment modalities are available for muscle injuries, including PRICE protocols, stretching, functional rehabilitation, physical therapies, but the best management is still debated in literature. Based on anatomy, biomechanics, and imaging features of muscle injury, a new classification system has been proposed. We provide an evidence-based overview on treatment protocols and surgical interventions for muscular injuries in athletes.

## Epidemiology

Muscle injuries usually occur in the eccentric phase of the muscle contraction after an indirect insult, more common in not contact sports, and after direct trauma, as in contact sports[3,4].

Traumatic lesions vary depending on the direction and angle of movement of the forces applied. When the trauma is direct, an external force is applied to the muscle, and external and internal structures are squeezed against each other. The injury therefore depends on the impact intensity, state of contraction of the muscle, traumatic moment, and muscle injured [5]. In indirect trauma, there is no external traumatic force, and the main cause of injury is an eccentric contraction of the muscle. The muscles most frequently involved are those containing a great percentage of type II fibres, with a pennate architecture, and crossing 2 joints: the hamstrings, rectus femoris, and medial head of the gastrocnemius [6]. The main site of injury is the musculotendinous junction [7]. In professional soccer, muscle injuries account for 31% of all injuries, and are responsible for 25% of days of absence away from training and competition. Most of these lesions (96%) are indirect, mostly to the hamstrings and rectus femoris; the adductor and the quadriceps may be involved, especially when the athlete attempts to kick the ball [8]. On the other hand, compared to other players, goal keepers are less susceptible to muscle injuries [9]. These injuries are less frequent in young athletes: the injury incidence is 1.19 per 1000 hours of training activity in soccer players younger than 22 years, and 1.63 for those older than 30 years. The incidence is 6.6 per 1000 hours of competition in younger athletes, and 9.5 in older soccer players [9].

Age predisposes to muscle injuries [10]. The triceps surae muscle is commonly injured in patients older than 40 who practice pivoting sport activities [11]. This is a consequence of aging related changes within the muscle, such as the re-arrangement of motor units and denervation. In an effort to substitute denervated muscle fibres, even though the residual motor units will hypertrophy, their capacity to finely regulate force intensity will be lower.

In other sports, the incidence of muscle injuries is variable: 11% in rugby, 16% in running sports, 18% in basketball. In these sports, the hamstrings, quadriceps and adductor muscles are the most frequently affected [12,13,14].

## Anatomy, biology, biomechanics

Skeletal muscles are composed of individual muscle cells known as myocytes, also called “muscle fibres” or myofibres. These are formed from the fusion of myoblasts to constitute long, cylindrical, multinucleated cells. Single muscle fibres are engrouped in fascicles surrounded by connective tissue, the perimysium; each fibre is surrounded by a single layer of connective tissue, the endomysium [15].

A motor unit is composed of an alpha motor neuron and the skeletal muscle fibres which its axon innervates. In terms of speed of contractions, muscle fibres may be slow (S) or fast (F). The latter may be fatigue resistant (FR) fibres, fast-twitch fatigable (FF), and fast contraction fibres with intermediate features (Fint).

The musculotendinous junction (MTJ) connects the skeletal muscle to its tendon, forming with the muscle a complex biomechanical unit. The MTJ is located at the extremities of the muscle fibres, where the fibres end, and merge with the tendon fibres. This is the main site in which the force produced by muscle contraction is transmitted [16]. In this region, the muscle enormously increases the contact area with the tendon, via deep interdigitations of the cell membrane, allowing the junction to resist the muscle contraction, from 1.8 to 3.5×10^4^ N/m^2^ [16]. Specifically, these interdigitations transmit the force of muscle contraction to the tendon fibres in a tangential direction, to transmit the tension developed to the joint and bone. Physical exercise may modify the architecture of the fibres, increasing the amount of interdigitations and the tension developed by each unit [17].

## Mechanism of muscle injury

A lesion occurs at the site of the impact when the trauma to the muscle is direct, at the MTJ, or at the end of the muscle belly when the trauma is indirect. The structural damage to the muscle fibres may be caused by a single contraction or by the cumulative effect of several contractions. An eccentric contraction is a major cause of injury, probably as consequence of the greater forces produced by eccentric contractions compared to isometric or concentric contractions [6,18]. When excessive, an eccentric contraction may be harmful, causing delayed onset muscle soreness, muscle strain, acute tendon injury, overuse tendinopathy, and tendon ruptures. However, eccentric stretching training may prevent the occurrence of injury of the muscle-tendon unit by increasing the ability of the muscle to absorb loads [19,20]. It also reduces the muscle stiffness and visco-elasticity of the muscle tendon system. It is important to consider the condition of the muscle during in the eccentric phase of the contraction: the fact that some muscles develop lower strength in shortened position, and normal strength when lengthened, supports that stretching protocols should be recommended before eccentric loading to prevent pain and loss of strength [21]. Structural changes and diminished capacity in the excitation contraction coupling mechanism may also be observed. Intensive training may prevent these traumatic changes, as it occurs when undertaking short repetitive eccentric contractions which evoke evident protective adaptations of the muscle. Since an eccentric contraction develops forces greater than those produced in isometric and concentric contractions, eccentric training may overload the muscle and increase significantly its power. However, this training has to be done at gradually increasing speed and against progressive loads.

## Classification

According to the mechanism of trauma, muscle injuries may be distinguished as direct and indirect.

### 

#### Injury after direct trauma

A contusion is an insult from a direct trauma against the opponent or a sport related tool; a laceration arises from an impact against a sharp surface. Lacerations are not classified further. Contusions may be classified as mild, moderate, and severe according to the functional disability which they produce. Athletes should be re-examined 24 hours after the trauma to better assess the entity of the injury, as the pain may be disabling at the time of the injury, and the lesion is overestimated.

#### Injury after indirect trauma

There is no impact against the opponent or any tool. These injuries are classified as non-structural and structural [22]. In non-structural injuries, the muscle fibres do not present an anatomically evident lesion; structural injuries present an anatomically defined lesion.

***Non-structural Injuries***: are the most common, accounting for 70% of all muscle injuries in soccer players [23]. Even though the lesion may not be readily recognized, they cause more than 50% of days of absence away from sport activity and training. When neglected, they may become structural injuries. A 1A injury is caused by fatigue and changes in training protocols, running surfaces, and high intensity activities. A 1B injury may result from excessive and prolonged eccentric contractions. A 2A injury is mainly associated with spinal disorders, often misdiagnosed, as in minor inter-vertebral disorders which irritate the spinal nerve, altering the control of the muscle tone of the “targeted” muscle. In these instances, the management of the spinal disorder is the primary target.

A 2B injury arises from an unbalanced control of the neuro-musculoskeletal system, mostly of the mechanism of mutual inhibition coming from the muscle spindles. An imbalance of these neuromuscular mechanisms may compromise the control of the muscle tone, and induce muscle disorders. This occurs when the inhibition system of the agonist muscles is altered (diminished), and the agonist muscle is excessively contracted for compensation.

***Structural injuries***: are divided into three sub-groups according to the entity of the lesion within the muscle. A type 3A lesion is a minor partial lesion involving one or more primary fascicles within a secondary bundle. A type 3B lesion is a moderate partial lesion involving at least a secondary bundle, with less than 50% of breakage surface. A type 4 lesion is a sub-total tear with more than 50% of breakage surface or a complete tear of the muscle, involving the muscle belly or the musculotendinous junction.

Our classification of structural injuries defines also the site of the lesions in terms of proximal (P), middle (M), and distal (D). The prognosis of proximal lesions of hamstring muscles and rectus femoris is worse than that injuries of same size involving the middle or distal portion of these muscles. In the triceps surae, distal injuries present the worst prognosis.

### Diagnosis

The diagnosis of muscle injury is mainly based on history and clinical examination. In a contusive injury, pain onset is usually immediate, the insult is direct, and symptoms increase in association with size and entity of the hematoma. The active range of motion is reduced, and the athlete cannot continue to train and compete. When functional disability appears early, a new assessment is recommended after 24 hours to better define the injury.

In non-structural injuries, athletes complain of soreness, heaviness and stiffness of the muscle, usually increasing with exercise, at times present at rest. On palpation, it is possible to appreciate stiffness of some bundles.

In delayed onset muscle soreness (DOMS) (Type 1B), pain usually occurs at rest, some hours after sport activity, and the whole muscle is stiff on palpation.

In 2B injuries, athletes report cramps; adequate stretching improves symptoms. At times, repeated fatigue related or neuromuscular disorders may indicate subclinical muscle pathologies, unmasked by intense loading training protocols. Intense prolonged exercises may damage the muscle: the serum levels of enzymes, markers of the functional status of the muscle, change after both physiological stimulations (exercise) and pathological conditions (myopathy) [24]. Increased levels of these markers may indicate cell necrosis and tissue damage related to intense acute or chronic muscle stress. Levels of creatin kinase (CK) should be monitored: they may increase acutely, after an intense stress to the muscle or following chronic insults, as in myopathy [25]. Serum CK levels are higher in athletes, a consequence of continuous strenuous stresses to the muscle [26]. Nevertheless, after rest, in the absence of trauma, drug administration and other pathologies, these levels are lower compared to those observed in sedentary controls [27]. Persistently high CK levels at rest may indicate a subclinical genetic muscle disorder which may be unmasked by training, with occurrence of fatigue, DOMS, and persistent contractures [28].

A minor partial lesion (Type 3A) is characterized by sharp pain, evoked by a specific movement. Pain is well localized, easy to appreciate on palpation and, at times, preceded by a snap sensation. On palpation, it is not possible to detect the structural defect as it is too small and the contraction against manual resistance is painful.

In a partial moderate lesion (Type 3B), acute pain, sharp, evoked by a specific movement. A snap may be appreciated, immediately followed by localized pain and functional disability, up to inducing the athlete to fall down. On palpation, pain is localized and the structural defect may be appreciated, with possible evidence of hematoma a few days later, especially when the epymisium or perimisium are involved. The estensibility test is positive, and contraction against resistance is usually impossible.

Subtotal/total ruptures or tendon avulsions (Type 4) present with dull, oppressive pain exacerbated by a specific movement; snapping and functional disability appear immediately. The interruption within the muscle may be palpated, and a hematoma develops early. The function of the MTJ is lost.

### Imaging

Ultrasound (US) is a first level diagnostic tool, cheap, fast to perform, which allows staging of almost all muscle lesions, and assessment of their evolution and complications. Magnetic Resonance Imaging (MRI) is needed in few instances as US does not allow to assess deeper tissues, to perform an early evaluation, and has low sensitivity for small lesions. Specifically, US sensitivity is 77% and 93% for non-structural and structural injuries, respectively. Reasonably, this is related to the fact that, in minor trauma, the diagnostic capability of US is poor, as there is little edema [1]. The sensitivity for mild contusions is slightly higher than for non-structural injuries. An atypical US feature is the presence of edema imbibition without evidence of fiber interruption, and a differential diagnosis is based on clinical history. In severe contusions, US sensitivity to assess the hematoma is 100% [29]. US allows to diagnose a structural injury of the muscle 36 to 48 hours after the trauma, as the peak of hemorrhagic edematous collection is observed after 24 hours, and until 48 hours, when it will start to decrease [29]. In professional and high level athletes, MRI may confirm or exclude minor structural injuries, when clinical examination and US are discordant. However, the most recent evidences recommend to combine MRI and US as MRI alone cannot precisely measure the extent of the structural damage [22]. US monitoring can be performed 2, 4, and 5 days after the trauma. Dynamic US examination allows to assess both elongation and dislocation of tertiary bundles, and the extent of the lesion [30].

Colour Doppler and Power Doppler US allow to visualize the course of arteries and veins, and quantify the amount of blood within the muscle. In this way, it is possible to depict the hypervascularity within the scar tissue of the lesion: this would indicate that the reparative scar tissue at the site of lesion is unstable.

MRI is a multi-parametric diagnostic tool. Differently from US, high intrinsic contrast and fluid sensitive (STIR and T2) MRI sequences allow to detect also minimal changes, with 92% sensitivity for non-structural injuries [31]. It is also panoramic, providing a wide evaluation of deeper muscles that are difficult to study at US. Structural lesions are well recognized at MRI. Axial scans, through a comparative examination, measure changes in volume, structure, and signal intensity of the muscle; coronal and sagittal scans along the axis of the belly define properly the extent of the lesion.

MRI is more sensitive than US to depict the signal changes arising from the presence of edema within the muscle bundle, even when the muscle is structurally intact [32]. MRI is therefore indicated 1) to provide a prognosis for non-structural injuries in professional and high level atlete; 2) to exclude a structural injury in professional high level athletes when clinics and US are discordant; 3) to assess muscles difficult to examine at US; 4) in subtotal or complete muscle lesions when suspecting tendon involvement or bone-tendon avulsions. Contrast medium MRI (Gadolinium) may be useful to monitor the stability of the scar tissue after a structural injury.

### Imaging features

#### Non-structural injuries

US is often negative; at times, transient hyperecoic or hypoecoic changes may appear (3–5 days); Power Doppler US does not allow to identify any negative features. This is also true at MRI, where, at times, there could be evidence of limited edema.

#### Structural injuries

Our classification is based on anatomy and extent of the lesion [33]. It may be difficult to differentiate mild from moderate partial injuries, especially when the lesion is small. Given the presence of liquid on MRI scans, MRI may overestimate the entity of the injury [22]. In acute mild partial injuries, US shows a slightly hyperechoic area which, later, becomes inhomogeneous and hypoechoic, focalized, with some structural disarray within which it is possible to detect a small anechoic area in the context of the muscle. At MRI, edema imbibition and mild inhomogeneous signal hyper-intensity may appear, because of the interstitial and peri-fascial edema or small hemorrhagic extravasation.

In acute lesions, the US appearance of a partial moderate lesion is characterized by a hyperechoic area which becomes markedly inhomogeneous, with evidence of structural disarray, and a wide anechoic area within and outside the muscle. At MRI, the muscle is enlarged because of edema imbibition, with inhomogeneous signal hyper-intensity related to interstitial and peri-fascial edema or hemorrhagic extravasation.

At US, subtotal or total lesions appear severely as inhomogeneous and disorganized iso- or hyper-echoic areas. Successively, it is possible to appreciate inhomogeneity and marked structural changes, retraction of the ends of rupture and wide anechoic area within and between muscles. At MRI, there is muscle end retraction, collection of hyper-intense fluid caused by hemorrhagic extravasation between the 2 muscle ends.

### Management

Most of muscle injuries respond well to conservative treatment, that should follow different phases (Table 1).

#### First phase: acute management

In the first 2–3 days after injury, local therapy using ice may be combined with moderate exercise (active and passive stretching) as tolerated. Athletes with severe lesions in the lower limb should walk with crutches, and avoid excessive lengthening of the injured muscle in the first 3–7 days after injury. The PRICE protocol (Protection Rest Ice Compression Elevation) is indicated in the early stages, once the structural lesion has been documented. The injured muscle has to be protected by excessive loads which could impair or slow the healing process down. Rest is necessary to reduce the metabolic request over the injury site and prevent the increase of hematoma and swelling. Ice (cryotherapy) is used to reduce the local temperature, the metabolic request, and bleeding. In addition, it also improves pain by inhibiting pain receptors and increasing the latency of conduction of nervous fibres. Compression limits edema diffusion from fluid extravasation from injured vessels within the site of lesion. In this way, controlling the increased inflammatory exudate, it would be possible to reduce the formation of not functional scar tissue and aid the homeostasis of interstitial fluids. Elevation of the injured area reduces local pressure and bleeding, promotes the drainage of inflammatory exudate through the linfatic system, and reduces edema and related complications [34]. We now promote the “POLICE” (Protection Optimal Load Ice Compression Elevation) protocol [35]. Optimal load is probably the only major innovation. In this way, the injured muscle will rest, but a balanced progressive rehabilitation program should gradually introduce controlled mechanical stresses, different according to the affected site, and athletic feats determined by involved muscles [35]. At this stage, manual therapy consisting of specific massages aiding the drainage of the tissues not compromised, close to the site of injury, may improve the disposal of inflammatory catabolites. Functional compressive bandages may also aid to reduce local pressure, improve pain, and optimize the effects of physiotherapy and rehabilitation. In this phase, low intensity laser therapy (LLLT), pulsed ultrasound therapy, and electroanalgesia may be used.

#### Second stage: Post-acute management (3 to 7 days after trauma)

Muscle stretching may be passive, assisted or active. There is no evidence that passive stretching is superior to an active protocol in terms of muscle lengthening and muscle flexibility. Neural mobilization (neurodinamics) may also aid detensioning of peripheral neural structures, and increase local flexibility of the muscle. Isometric training may also promote muscle recovery; concentric and eccentric exercises may be started when moderate isometric training can be performed without pain. Initially, these exercises should be undertaken without resistance, and the load should be progressively increased [18]. Isotonic eccentric loads should follow concentric training. These protocols must be administered in the absence of pain, respecting the healing process, and time of recovery. Methods of neuromuscular bandage have been introduced in the last decades. The rationale is to reduce the tension over the injury site by detaching the skin from subcutaneous and deeper tissues: the analgesic effect related to the draining process on these tissues should improve edema and swelling. Sensorial motor training consists of balance exercises on stable or unstable surfaces, different in size and shape, with or without request of additional cognitive tasks, with or without the support of vision. It is possible to include core stability exercises to improve postural and neuromuscular control. This training could be also helpful to prevent the occurrence of recurrences. Among instrumental physical therapies, endogenous termotherapy, high intensity laser therapy (HLLT) and continuous ultrasound therapy may also be used at this stage. On the other hand, the role of hydrotherapy is still uncertain.

#### Third stage: Functional rehabilitation and general athletic reconditioning

This is a sport specific rehabilitation stage which involves the metabolic system, specific and individualized training protocols, fitness and strength training. It is a multi modular approach which aims to improve sensitive and motor abilities, muscle resistance and strength [36]. Isokinetic and complex multi task exercises (including cognitive tasks) are started [36].

#### Fourth stage: Athletic reconditioning and specifical strength

It is possible to start high intensity training protocols based on strength, athletic reconditioning, and sport specific abilities [37]. Pliometric, ballistic and isonertial exercises are started. At the end of this stage, the athlete should be able to repeat many series of the sport specific movements which had caused the traumatic insult.

#### Fifth stage: return to competition

The athlete may gradually return to full activity and follow rehabilitation training to prevent recurrences or the occurrence of new injury.

### Surgical treatment

The main indication to surgery are complete lesion of the muscle belly or of the MTJ, and subtotal lesion associated with persistent pain and loss of strength after conservative management [38]. Muscle laceration repair is technically demanding and the likelihood of clinical failure is high. Muscle belly is difficult to repair successfully because the sutures pullout. Many suture configurations have been described, but there is no clear advantage of one over another. Suture techniques can be divided into conventional, such as Kessler stitches, horizontal mattress, and figure of eight stitches, and complex. Modified Kessler suture, modified Mason-Allen suture, combination stitch and muscle suture with augmentation belong to the second group [39]. Optimal suturing of muscles should permit early rehabilitation with a low risk of rerupture or stitch pullout. The present trend is to use configurations resistant to traction and tensile loading, with lower risk of pullout [38]. Incorporation of the epimysium significantly improves the biomechanical properties of sutured muscle bellies [40]. Even though excellent results have been reported after repair of complete lesions, the evidence in favor of routine surgical management is still scanty.

### Future Perspectives

Platelet-rich plasma products are now widely tested in different fields of medicine. They contain a higher concentration of platelets than that of the blood. Platelets contain dense and alpha granules, involved in tissue modulation and regeneration and induce a cascade which leads to the healing process, which occurs through three phases: inflammation, proliferation, and remodeling [41]. PRP products may promote healing, but the exact mechanism through which it occurs is still unclear, and the evidence is still of low quality. Given our rudimentary knowledge of the mechanism of action of the PRPs, we prompt researchers to undertake appropriately powered level I studies with adequate and relevant outcome measures and clinically appropriate follow up. Recent studies have reported encouraging results combining PRP and Losartan, an antihypertensive drug which acts against the process of fibrosis after muscle injury, and promotes healing by stimulating regeneration and angiogenesis. Losartan seems to be very effective if administered not immediately, but 3 to 7 days after the injury [42].

## Conclusion

Diagnosis is of muscle injury is mainly based on history and clinical examination, but imaging is useful to classify and to provide the prognosis of the lesion. Most muscle injuries are treated conservatively with excellent results. A step-by-step treatment protocol is fundamental to achieve good results and reduce reinjuries. Complete lesions of the muscle belly or of the MTJ, and subtotal lesion associated with persistent pain and loss of strength after conservative management require a surgical intervention.
